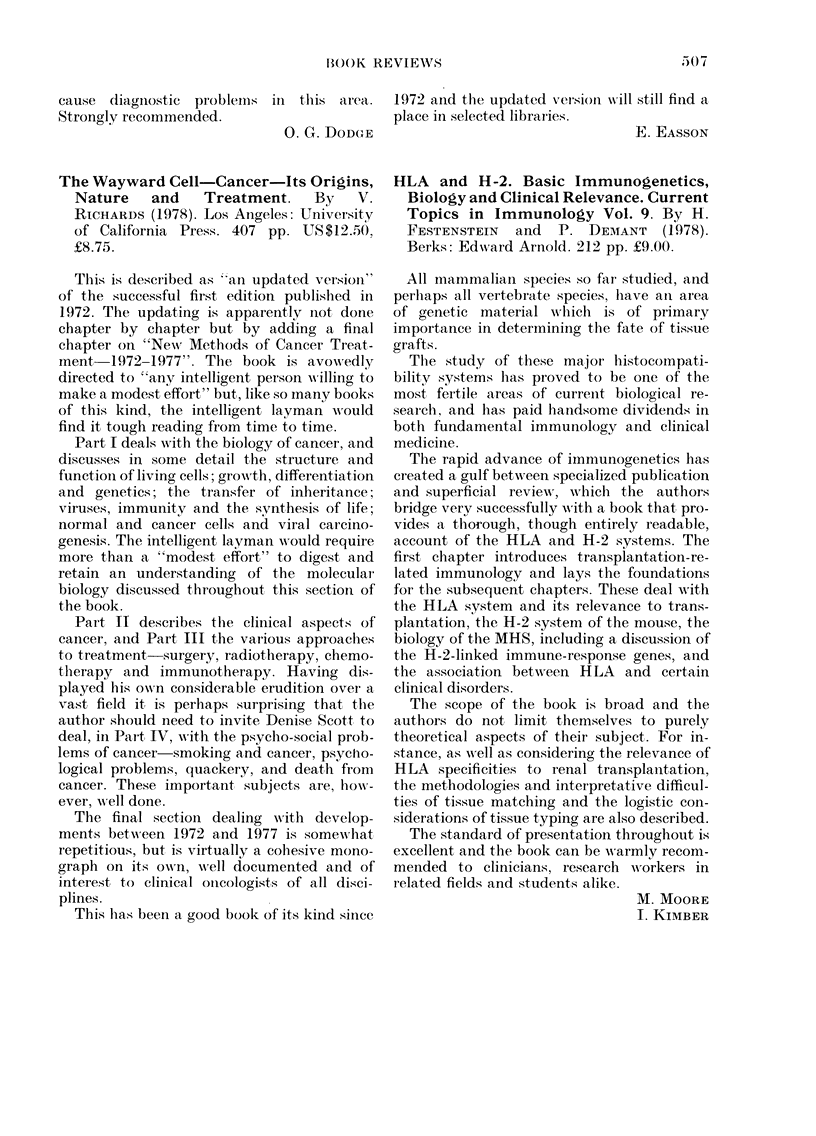# The Wayward Cell—Cancer—Its Origins, Nature and Treatment

**Published:** 1979-09

**Authors:** E. Easson


1972 and the updated version will still find a place in selected libraries.

E. EASSON

**The Wayward Cell—Cancer—Its Origins, Nature and Treatment**. By V. RICHARDS (1978). Los Angeles: University of California Press. 407 pp. US$12.50, £8.75.

This is described as "an updated version" of the successful first edition published in 1972. The updating is apparently not done chapter by chapter but by adding a final chapter on "New Methods of Cancer Treatment—1972–1977". The book is avowedly directed to "any intelligent person willing to make a modest effort" but, like so many books of this kind, the intelligent layman would find it tough reading from time to time.

Part I deals with the biology of cancer, and discusses in some detail the structure and function of living cells; growth, differentiation and genetics; the transfer of inheritance; viruses, immunity and the synthesis of life; normal and cancer cells and viral carcinogenesis. The intelligent layman would require more than a "modest effort" to digest and retain an understanding of the molecular biology discussed throughout this section of the book.

Part II describes the clinical aspects of cancer, and Part III the various approaches to treatment—surgery, radiotherapy, chemotherapy and immunotherapy. Having displayed his own considerable erudition over a vast field it is perhaps surprising that the author should need to invite Denise Scott to deal, in Part IV, with the psycho-social problems of cancer—smoking and cancer, psychological problems, quackery, and death from cancer. These important subjects are, however, well done.

The final section dealing with developments between 1972 and 1977 is somewhat repetitious, but is virtually a cohesive monograph on its own, well documented and of interest to clinical oncologists of all disciplines.

This has been a good book of its kind since